# Premature ovarian insufficiency in female adolescent and young adult survivors of non-gynecological cancers: a population-based cohort study

**DOI:** 10.1186/s12978-022-01559-8

**Published:** 2023-01-02

**Authors:** Sydney B. Flatt, Amanda Baillargeon, Chad McClintock, Jessica Pudwell, Maria P. Velez

**Affiliations:** 1 School of Medicine, Queen’s University, 15 Arch St., Kingston, ON K7L 3L4 Canada; 2 Department of Obstetrics and Gynecology, Queen’s University, 76 Stuart St., Victory 4, Kingston, ON K7L 2V7 Canada; 3 ICES Queen’s, 21 Arch St, Kingston, ON K7L 2V7 Canada

**Keywords:** Premature ovarian insufficiency, Hematologic cancer, Breast cancer, Thyroid cancer, Young adults

## Abstract

**Background:**

The risk of premature ovarian insufficiency (POI) is increased in adolescent and young adult (AYA) cancer survivors, with the prevalence depending on cancer diagnosis, treatment, and patient factors. Prior studies are limited by sample size and type of cancer included. The objective of this study was to assess the risk of POI in female AYA survivors of non-gynecologic cancers, using a population-based approach.

**Methods:**

This population-based retrospective cohort study comprises 21,666 females, 15–39 years old, diagnosed with a single non-gynecologic cancer in Ontario, Canada from 1995 to 2015.

Through health administrative data linkage, participants were followed until their 40th birthday, December 31, 2018, bilateral oophorectomy, loss of health insurance eligibility or death. Each cancer survivor was matched to 5 females who were not diagnosed with cancer (unexposed, n = 108,330). Women with bilateral oophorectomy or a prior menopause diagnosis were excluded. POI was identified through use of the ICD-9 code for menopause (ICD9-627). Modified Poisson regression models were used to calculate the adjusted relative risk (aRR) of POI for AYA cancer survivors compared to unexposed individuals, adjusted for income, parity, age, and immigration status.

**Results:**

The occurrence of POI was higher in survivors of AYA cancer versus unexposed patients (5.4% vs. 2.2%). Survivors of AYA cancer had an increased risk of POI relative to unexposed patients (aRR 2.49; 95% CI 2.32–2.67). Risk varied by type of cancer: breast (4.32; 3.84–4.86), non-Hodgkin’s lymphoma (3.77; 2.88–4.94), Hodgkin’s lymphoma (2.37; 1.91–2.96), leukemia (14.64; 10.50–20.42), thyroid (1.26; 1.09–1.46) and melanoma (1.04; 0.82–1.32). Risk varied by age at time of cancer diagnosis, with a higher risk among females diagnosed at age 30–39 years (3.07; 2.80–3.35) than aged 15–29 years (1.75; 1.55–1.98).

**Conclusions:**

AYA survivors of non-gynecologic cancers are at an increased risk of POI, particularly survivors of lymphomas, leukemia, breast, and thyroid cancer. The risk of POI is increased for those diagnosed with cancer at an older age. These results should inform reproductive counseling of female AYAs diagnosed with cancer.

## Plain language summary

Premature ovarian insufficiency is the onset of premature menopause in individuals less than 40-years-old. Previous research has shown that there is a higher risk of premature ovarian insufficiency in adolescent and young adult cancer survivors, due to the toxicity of cancer treatments on reproductive organs. Prior research was limited in its applicability by having small sample sizes, only including childhood cancer, excluding young adults, and studying fewer types of cancer. This study was conducted using a large population-based approach, on all females aged 15–39 years old with cancer in Ontario, Canada from 1995 to 2015. We found that there was nearly a 2.5 times greater risk of premature ovarian insufficiency in cancer survivors compared patients without cancer. Compared to patients without cancer, this risk was highest for survivors of leukemia (14 times higher risk), followed by breast cancer (4 times higher risk), lymphomas (2–4 times higher risk), and thyroid cancer (1.2 times higher risk). There is no increased risk in melanoma survivors. The risk was higher in individuals diagnosed with cancer at a later age (30–39 years), with a risk 3 times higher than the population without cancer, while a younger age of diagnosis (15–29 years) carries a risk only 1.75 times higher than the population without cancer. These results should help improve healthcare provider and patient understanding of the risk of premature ovarian insuficiency in young cancer survivors, and guide counseling at the time of cancer diagnosis and during survivorship on future reproductive function.

## Introduction

The survival rate of adolescents and young adults (AYAs), aged 15–39, diagnosed with cancer, has now reached > 80% [1]. AYA survivors are more likely to live beyond their diagnosis and treatment compared to their late adult-onset counterparts; thus, the long-term impact of cancer is disproportionately greater in this population [1]. Consequently, the long-term quality-of-life implications of cancer treatments must receive increasing consideration. Studies thus far demonstrate an increased risk of premature ovarian insufficiency (POI) in AYA cancer survivors [2]. POI is the loss of ovarian activity before age 40, defined as the presence of amenorrhea for ≥ 4 consecutive months and biochemical confirmation of hypergonadotropic hypogonadism (FSH > 25 or 30 IU/L, depending on the guideline, 1 month apart) [3]. The implications of POI are extensive, including long-term impacts on fertility, sexual dysfunction, cardiovascular disease, osteoporosis, cognitive function, mental health, quality-of-life, and premature mortality [3].

Research suggests a prevalence of POI ranging from 2.1 to 82.2%, in survivors of pediatric and AYA cancer, depending on cancer diagnosis, treatment, and patient factors. [2] Strong evidence demonstrates an increased risk of POI in those treated with alkylating agents and radiotherapy to the abdomen and pelvis, particularly at higher doses [4–8]; however, given that most of these studies were conducted in cohorts treated over 30 years ago, little is known about the impact of contemporary protocols. Furthermore, studies thus far have focussed largely on cancer survivors < 21 years old, neglecting patients 21–39 years old, who are also impacted during their reproductive years [4, 5, 9–14]. Studying the AYA population is especially important as evidence shows that cancer survivors treated after pubertal onset with alkylating agents or low-dose ovarian radiation had, respectively, a 9- and 29-fold higher rate of POI, compared to survivors treated before pubertal onset [13]. In addition to treatment gonadotoxicity, timing and duration of ovarian function depends on age at treatment. Younger age at treatment is associated with higher trajectories of reproductive function, but the protective effect of younger age is not observed in survivors exposed to highly gonadotoxic treatments [15]. Thus, the objective of this study was to assess the risk of POI in female AYA survivors of non-gynecologic cancers, using a population-based approach in Ontario, Canada from 1995 to 2015.

## Methods

A retrospective population-based cohort study was conducted using linked administrative healthcare databases through ICES (www.ices.on.ca) in Ontario, Canada. ICES is an independent, non-profit research institute funded by an annual grant from the Ontario Ministry of Health and the Ministry of Long-Term Care. As an entity under Ontario’s privacy legislation, ICES is authorized to collect and use health care data for the purposes of health system analysis, evaluation, and decision support. Secure access to this data is governed by policies and procedures that are approved by the Information and Privacy Commissioner of Ontario.

### Data collection

Data regarding incident cancers were obtained from the Ontario Cancer Registry (OCR), a provincially mandated registry containing information on all cancer diagnoses in Ontario since 1964. The OCR is over 95% complete [16]. The Ontario Health Insurance Plan (OHIP) database was also used, which contains physician billing claims for services, allowing for the identification of nearly all medical consultations and diagnoses. OHIP is a government run and funded healthcare plan, which provides universal health coverage to all residents of Ontario, the largest province in Canada, for medically necessary services. The Registered Persons Database (RPD) was used to identify demographic and eligibility information on OHIP recipients. The Immigration Refugees and Citizenship Canada Permanent Resident (IRCC-PR) database was used to identify information about immigrations status. The MOMBABY database was used as a validated database of pregnancy outcomes and mother-infant linkage to identify patients’ parity. MOMBABY contains information on all pregnancies > 20 weeks gestation since 1988, resulting in a hospital livebirth, stillbirth, or pregnancy termination, and captures approximately 98% of all births in Ontario [17]. Women were classified as parous if they had a history of delivery prior to their cancer diagnosis or the enrollment date. Socioeconomic status was determined using the income quintile associated with the census dissemination area of the residence, at the date of cancer diagnosis or enrollment date. Rurality was determined using the Rurality Index for Ontario (RIO), with rural being defined as an RIO ≥ 40, and urban defined as an RIO between 0 and 39. POI, the outcome of interest, was identified through physician billing claims for menopause prior to age 40 years via the OHIP database (ICD-9 code 627). This is a diagnostic code used by the providing physician for service compensation. Internal validation of the OHIP billing code ICD-9 627 against FSH levels > 25 IU/L available in a subset of this cohort, demonstrated low sensitivity 30.1% (95% CI 29.1–31.2), but high specificity of 97.0% (95% CI 97.0–97.1).

### Study population

**Fig. 1:**
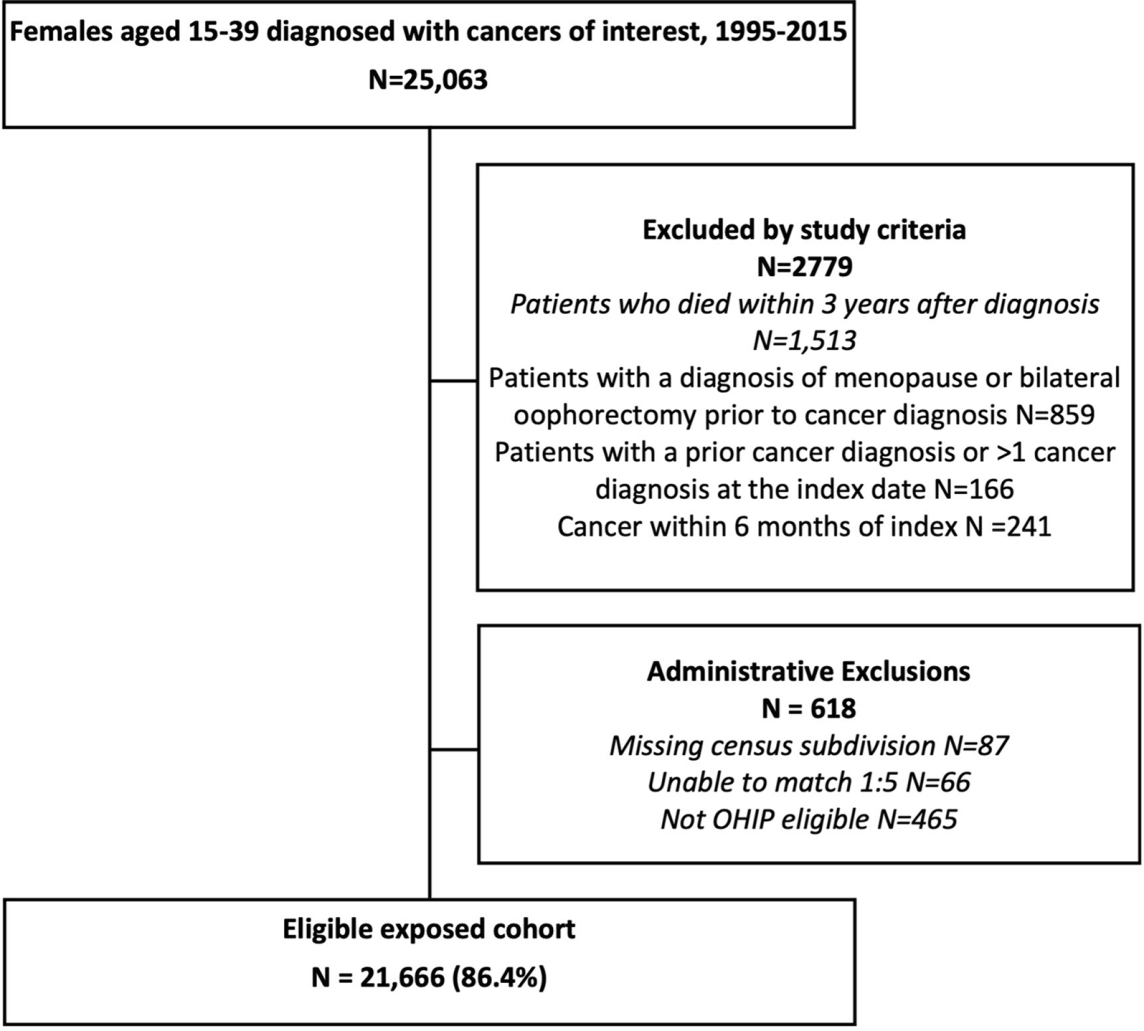
Study flow chart

All females aged 15–39 years old at the time of diagnosis of a single non-gynecological cancer, from January 1, 1995, to December 31, 2015, were identified (n = 25,063) (Fig. 1). We assessed the most common non-gynecological cancers in this population, including leukemia, breast cancer, non-Hodgkin lymphoma (NHL), Hodgkin lymphoma (HL), and thyroid cancer [1]. Excluded were those who died within 3 years after diagnosis, had a diagnosis of POI prior to cancer diagnosis, history of bilateral oophorectomy, any history of prior cancer diagnosis or subsequent cancer within 6 months of index diagnosis, or those who had missing geographical census data in ICES. This resulted in an exposed cohort of n = 21,666 (Figure 1).

Each exposed patient (cancer survivor) was matched by birth year and geographic region of residence based on census subdivision to 5 individuals without a cancer diagnosis (unexposed), randomly selected without replacement (n = 108,330). The index date for each unexposed individual was assigned as the diagnosis date of the matched survivor. Unexposed patients were excluded if they were missing census subdivision data, died within 3 years of the index date, or had a diagnosis of cancer recorded in the OCR prior to the enrollment date. Individuals were followed until their 40th birthday, December 31, 2018, bilateral oophorectomy, loss of OHIP eligibility, subsequent cancer diagnosis, or death.

### Statistical analysis

Analyses were conducted using SAS version 9.4 (Cary, North Carolina) at ICES Queen’s. Baseline patient characteristics were compared using standardized differences, where a value greater than 0.10 is considered a clinically meaningful imbalance. [18] The standardized difference (SD) describes differences between groups in units of standard deviation; therefore, it is not influenced by sample size and is a better alternative to the p-value in large cohorts. Modified Poisson regression models, accounting for matched pairs and follow-up time, were used to calculate the adjusted relative risk (aRR) of POI for AYA cancer survivors relative to unexposed individuals, adjusted for income quintile, parity, age, and immigration status. Given that a distinct spectrum of diseases is seen for age groups 15–29 and 30–39 [1], we performed pre-planned stratified analyses by these 2 age groups.

### Ethical approval

This study was approved by the Queen’s University Health Sciences and Affiliated Teaching Hospitals Research Ethics Board.

## Results

### Patient characteristics

Patient characteristics of AYA cancer survivors were similar to unexposed individuals (Table 1). No differences existed in age at cohort entry, parity, immigration status, rurality or income quintile. The mean age at cancer diagnosis was 32.1 (SD 6.0) years. Of all AYA cancer survivors, a total of 8153 (37.6%) had thyroid cancer, 7064 (32.6%) had breast cancer, and 2953 (13.6%) had melanoma. Other types of cancer occurred less commonly.

**Table 1:** Characteristics of AYA cancer survivors and matched cancer-free individuals (unexposed)

Patient characteristics at diagnosis of cancer	Exposed N = 21,666	Unexposed N = 108,330	Standardized difference*
Age (years), mean ± SD	32.12 ± 5.95	32.14 ± 5.97	0
Age categories			
< 30 years	6168 (28.5)	30,798 (28.4)	0
≥ 30 years	15,498 (71.5)	77,532 (71.6)	0
Income quintile			
1	3927 (18.1)	21,699 (20.0)	0.05
2–4	13,336 (61.6)	66,487 (61.4)	0
5	4403 (20.3)	20,144 (18.6)	0.04
Rurality			
Rural	1927 (8.9)	9635 (8.9)	0
Urban	19,739 (91.1)	98,695 (91.1)	0
Immigration status			
Immigrant	4399 (20.3)	24,358 (22.5)	0.05
Non-immigrant	17,267 (79.7)	83,972 (77.5)	0.05
Parity			
Nulliparous	11,107 (51.3)	58,455 (54.0)	0.05
Parous	10,559 (48.7)	49,875 (46.0)	0.05
Type of cancer			
Breast cancer	7064 (32.6)	35,320 (32.6)	0
Leukemia	586 (2.7)	2930 (2.7)	0
Hodgkin lymphoma	1647 (7.6)	8235 (7.6)	0
Non-Hodgkin lymphoma	1263 (5.8)	6315 (5.8)	0
Thyroid cancer	8153 (37.6)	40,765 (37.6)	0
Melanoma	2953 (13.6)	14,765 (13.6)	0

All data are presented as n (%) unless otherwise specified

^*^A standardized difference greater than 0.10 is considered clinically significant

### POI risk

Mean age at cancer diagnosis and at POI diagnosis are presented in Table 2. The proportion of POI was significantly higher in AYA cancer survivors (5.4%) compared to unexposed individuals (2.2%). The proportion of POI ranged from 2.8% in thyroid cancer and melanoma survivors to 21.0% leukemia survivors. After adjusting for age, income, parity before cancer diagnosis, and immigration status, the aRR of POI for AYA cancer survivors was 2.49 (95% CI 2.32–2.67). Differences existed between types of cancer diagnoses. AYA cancer survivors of leukemia (aRR 14.64, 95% CI 10.50–20.42), breast cancer (aRR 4.32, 95% CI 3.84–4.86), NHL (aRR 3.77, 95% CI 2.88–4.94), HL (aRR 2.37, 95% CI 1.91–2.96), and thyroid cancer (aRR 1.26, 95% CI 1.09–1.46) had an increased risk of POI compared to unexposed individuals. No increased risk of POI was found between unexposed individuals and AYA cancer survivors with melanoma.

**Table 2:** AYA cancer and risk of premature ovarian insufficiency in Ontario, Canada

Type of cancer	Age at cancer diagnosis	Age at POI diagnosis	POI Rate	Unadjusted	Adjusted^a^
	Mean (SD)	Mean (SD)	n (%)	RR (95% CI)	RR (95% CI)
Unexposed	–	–	2434 (2.2)	–	–
All	32.1 (6.0)	34.8 (4.4)	1160 (5.4)	2.51 (2.33, 2.70)	2.49 (2.32, 2.67)
Breast cancer	35.1 (3.8)	36.5 (2.7)	502 (7.1)	4.41 (3.91, 4.96)	4.32 (3.84, 4.86)
Leukemia	29.2 (7.4)	31.7 (5.3)	123 (21.0)	14.50 (10.42, 20.18)	14.64 (10.50, 20.42)
Hodgkin lymphoma	26.0 (6.7)	31.4 (5.6)	124 (7.5)	2.38 (1.92, 2.97)	2.37 (1.91, 2.96)
Non-Hodgkin lymphoma	30.9 (6.5)	35.1 (4.0)	95 (7.5)	3.91 (2.97, 5.16)	3.77 (2.88, 4.94)
Thyroid cancer	31.5 (5.9)	34.9 (4.3)	232 (2.8)	1.26 (1.09, 1.47)	1.26 (1.09, 1.46)
Melanoma	31.4 (5.8)	34.6 (4.2)	84 (2.8)	1.04 (0.82, 1.33)	1.04 (0.82, 1.32)

^a^Adjusted for age, income, parity before cancer diagnosis, and immigration status

The risk of POI varied by age group (Table 3). The aRR of POI for age group 15–29 years was 1.75 (95% CI 1.55, 1.98), while in those 30–39 years the aRR was 3.07 (95% CI 2.80, 3.35). In both age groups, survivors of leukemia, breast cancer, NHL, and HL had an increased risk of POI, although the RRs were higher in the age group 30–39 than in those 15–29 years. Thyroid cancer was associated with POI in the group 30–39 years, but not in those 15–29 years. No increased risk of POI was found in melanoma survivors in either age groups.

**Table 3:** Adjusted relative risks of POI stratified by age group 15–29 years and 30–39 years compared to matched cancer-free individuals, overall and by cancer type

Type of cancer	Age 15–29 years	Age 30–39 years
	Adjusted^a^ RR (95% CI)	Adjusted^a^ RR (95% CI)
All	1.75 (1.55, 1.98)	3.07 (2.80, 3.35)
Breast cancer	2.10 (1.48, 2.98)	4.83 (4.25, 5.48)
Leukemia	14.22 (8.76, 23.08)	18.51 (11.05, 30.99)
Hodgkin lymphoma	2.03 (1.56, 2.66)	3.40 (2.32, 4.98)
Non-Hodgkin lymphoma	2.75 (1.80, 4.18)	4.78 (3.37, 6.77)
Thyroid cancer	1.15 (0.93, 1.44)	1.35 (1.11, 1.65)
Melanoma	0.97 (0.68, 1.39)	1.10 (0.79, 1.54)

^a^Adjusted for age, income, parity before cancer diagnosis, and immigration status

## Discussion

### Main findings

Our study demonstrates that female AYA survivors of non-gynecologic cancer have an increased risk of subsequent POI diagnosis, compared to unexposed individuals. The risk of POI is elevated in survivors of leukemia, breast cancer, NHL, and HL in both age groups, and thyroid cancer in the older group (30–39 years). No increased risk was identified in melanoma survivors. Moreover, our study demonstrates that the risk of POI varies by age, with a higher risk in the age group 30–39 years than in those 15–29 years.

### Interpretation

In comparison to our exposed cohort of 21,666 patients, the largest previous study thus far included only 2819 eligible childhood cancer survivors [5]. Additionally, other studies in the field have included much narrower age ranges, with most studies thus far focusing on cancer survivors < 21 years old, neglecting patients 21–39 years old [4, 5, 9–14]. The inclusion of young adults in this study was imperative, as several studies have revealed that an older age at cancer diagnosis is a risk factor for the development of POI [4, 9, 13], although one study found no association [8]. On the other hand, using data from the California Cancer Registry reported the opposite relationship, such that the risk of experiencing early menopause was increased at a younger age of cancer diagnosis in patients with HL, NHL and gastrointestinal malignancies [19]. However, this was only true for patients whose menses resumed within one year of diagnosis. The risk of experiencing acute ovarian failure was increased at an older age at diagnosis. In relation to the impact of age at cancer diagnosis, our study corroborates the former research, as we have demonstrated an increased risk of POI in young adults (age 30–39) compared with those aged 15–29 years. This increased risk is maintained across all types of cancer studied, excluding melanoma, with a higher risk of POI at an increased age of diagnosis for survivors of leukemia, breast cancer, NHL, HL, and thyroid cancer. Our study also demonstrates that this risk is applicable beyond the comparison of cancer treatment before and after pubertal onset, used in previous studies [4, 13], as 96% of girls achieve menarche by age 15 [20], the youngest age in our cohort. As well, our study supports the finding of the Reproductive Window Study, a cross-sequential study of the ovarian function of 763 female AYA survivors in California and Texas, which reported that younger age at cancer treatment was associated with higher long-term trajectories of ovarian function as measured by levels of the anti-Müllerian hormone (AMH) [15].

The results of this study may need to be interpreted differently than other studies in the field, based on the use of varying methods to define a POI diagnosis. While we identified a diagnosis of POI based on physician billing codes for menopause (ICD-9 627) in women < 40 years, other studies used different definitions. In the Childhood Cancer Survivor Study, the largest prior analysis in the field, self-report questionnaires were used to identify women with POI [5]. Here, patients were considered menopausal if they failed to experience spontaneous menses for a minimum of 6 months and other causes such as pregnancy and injectable hormone use were excluded. This study reported a prevalence of non-surgically induced POI of 8%, and a rate ratio of 13.21 for survivors of various childhood cancers, compared to controls [5]. This higher prevalence and risk may be explained by response biases in self-reported data. Several other studies also relied on self-reporting and questionnaires to identify women with POI. These included a study based in Ontario, Canada, which identified a prevalence of 8.8% [4], a US study which identified a prevalence of 31.42% [9], a small study of childhood sarcoma survivors which identified a prevalence of 49% [21], and a French study which identified a prevalence of 2.1% [13]. Importantly, these studies included patients diagnosed as early as 1945 and no later than 1998, thus reflecting older treatments that have changed considerably over time. Other smaller studies have relied on hormone measurements, including serum FSH levels, to diagnose POI. These studies tended to report a higher prevalence of POI, with values of 17% in a 100-participant large prospective Danish cohort [12], 31.25% in a study of 32 British participants with a history of HL [10], and 57.1% in a cohort of 21 participants in France who received high-dose chemotherapy and autologous bone marrow transplantation without radiation [11] . One larger prospective study with 921 participants from the St. Jude Lifetime Cohort also used serum FSH levels to diagnose POI, and found a prevalence of 10.9% for survivors of various childhood cancers [8].

Our study identified a prevalence of POI in AYA survivors of leukemia of 21%, while it was 7.5% in survivors of HL and NHL. In a case–control study of 2819 survivors of childhood cancer from the multicenter Childhood Cancer Survivor Study, Sklar et al. found that in survivors of childhood leukemia the occurrence of self-reported POI through survey data was 14% for leukemia, 56% for HL, and 3% for NHL [5]. In AYA survivors, using the California Cancer Registry, Letourneau et al., conducted a retrospective survey study of 1041 women diagnosed with cancer between the ages of 18 and 40 years, which identified an early menopause (< 45 years) prevalence of 37% (age 20) and 16% (age 35) for HL and 56% (age 20) and 16% (age 35) for NHL survivors [19]. These differences may be attributed to the use of self-reported data to ascertain a diagnosis of POI, and selection bias in prior survey studies (i.e., patients with POI might have been more interested in responding the survey than patients without POI). In addition, Letourneau et al. included patients’ with early menopause which will result in a higher proportion of patients meeting this diagnosis [19]. Differences among studies can also be explained by different treatment protocols in pediatric versus the AYA population, and therapies with different gonadotoxic potential.

Our study also identified that AYA survivors of thyroid cancer have an increased risk of POI. This differs from previous literature which found no difference in the age at menopause, up to 47 years old, between differentiated thyroid cancer survivors and controls [22]. Differences with our study may be attributed to the older study population, sole inclusion of treatment with radioiodine-131, and use of older data. These discrepancies should be evaluated in future research. In fact, in our population-based study on the risk of infertility in survivors of AYA cancer in Ontario we identified an increased risk of infertility in AYA survivors of thyroid cancer (1.20, 95% CI 1.10, 1.30) compared with an age-matched cohort of individuals without cancer [23]. As well, others have reported a decreased overall pregnancy rate in women with thyroid cancer (SIR 0.79; 95% CI 0.72, 0.86) and a decreased cumulative incidence of first pregnancy in nulliparous women (HR 0.69; 95% CI 0.59, 0.81) [24]. The impact of thyroid cancer on reproductive function needs further investigation. Finally, our study identified no increased risk of POI in survivors of melanoma. Nonetheless, our prior research has identified a small increased risk of infertility in melanoma survivors (RR 1.17, 95% CI 1.01–1.35), thus further research is needed on the reproductive impact of female patients with melanoma [23].

Overall, our findings highlight an increased risk of POI in AYA cancer survivors, contributing to the growing body of research underlining reproductive health after cancer. The ovarian reserve plays an important role in the physiopathology of POI after specific cancer diagnosis. The ovarian reserve declines with age, as documented by studies modeling the trajectories of AMH levels throughout the reproductive lifespan [25, 26]. Age at time of treatment, and hence ovarian reserve, will determine POI. For breast cancer and hematological malignancies, systemic cancer therapy, and pelvic radiation contribute the most to POI. For thyroid cancer, if an association with POI is further confirmed, the mechanisms need to be investigated given that thyroid cancer is not generally treated with chemotherapy, or radiotherapy to a field that involves the ovaries. These results should be incorporated into the counseling of female AYA cancer survivors by their primary care providers, cancer care providers, and fertility specialists, to improve patient understanding about their risk of experiencing POI and its health implications. Counseling should include a discussion on the risks of infertility and prompt referral for fertility preservation prior to treatment initiation if desired. These recommendations support the statements by current clinical practice guidelines [27–29]. In particular, the American Society of Clinical Oncology (ASCO) guidelines have evolved from recommending infertility risk discussion with cancer patients in 2006 [30], to emphasizing the importance of addressing gonadotoxicity and fertility preservation in all patients with reproductive potential, including the pediatric population in 2013 [31], to providing current guidance regarding fertility preservation options for people with cancer anticipating treatment in 2018 [27]. Yet, fertility preservation counseling and referral rates remain low. [32, 33] Thus, our research further advocates for increased efforts in knowledge translation and improvements in interdisciplinary coordination to overcome barriers to fertility preservation referral, as well as long-term surveillance of reproductive function in survivors of AYA cancer. In terms of surveillance, assessment of pre-treatment ovarian function, in particular through AMH levels, in premenopausal women with a diagnosis of breast cancer or haematological malignancy is recommended to predict post-treatment recovery of ovarian function [28].

### Strengths and limitations

Strengths of this study include the population-based study design, large sample size, and inclusion of adolescents and young adult cancer survivors. Study limitations included possible nondifferential misclassification of the study outcome. Such misclassification is likely to result in an attenuation of our risk estimates. In fact, when validated against FSH levels > 25 IU/L available in a subset of this cohort, the use of a single ICD-9 627 code as a diagnosis of POI, resulted in low sensitivity 30.1% (95% CI 29.1–31.2), but high specificity of 97.0% (95% CI 97.0–97.1). It is therefore likely that some misclassification resulted in an underestimate of the effect size. On the other hand, ovarian function may be lost directly following cancer treatment, which is a separate entity termed Acute Ovarian Failure (AOF), and can be subsequently restored [34]. Data suggests that much of the recovery occurs early on, with up to 50% having resumption of menses 12-months following treatment [34]. For most of the cancers included in our study the mean age at cancer diagnosis and at POI diagnosis differed by at least 3 years, except for Leukemia (2.5 years), and breast cancer (1.4 years), decreasing the likelihood of AOF being the diagnosis instead of POI. Another limitation was the inability to account for other potential confounding variables including family history of POI, ethnicity, and smoking status, as they are not available in the administrative databases used. Finally, this study did not evaluate the impact of specific cancer treatments (e.g., alkylating agents, immunotherapy) or medications to decrease the impact of gonadotoxic treatment (e.g., GnRH agonists), which were not recorded in the databases included in our study. Future studies in the AYA population are needed in this regard.

## Conclusion

In conclusion, there is a significantly increased risk of POI in female AYA survivors of non-gynecologic cancers, particularly leukemia, breast cancer, NHL, and HL. This risk is increased for patients diagnosed with cancer at an older age. Thyroid cancer might be a risk factor for POI, particularly in young female adults (30–39 years). These results will help guide reproductive counseling of female AYAs diagnosed with cancer, as they provide objective rates and risks of subsequent POI diagnoses at a population level.

## Data Availability

The data set from this study is held securely in coded form at ICES. Although data-sharing agreements prohibit ICES from making the data set publicly available, access may be granted to those who meet prespecified criteria for confidential access, available at www.ices.on.ca/DAS. The full data set creation plan and underlying analytic code are available from the authors upon request, understanding that the computer programs may rely upon coding templates or macros.

## Abbreviations

POI: Premature ovarian insufficiency
AYA: Adolescent and young adult
aRR: Adjusted relative risk
OCR: Ontario Cancer Registry
OHIP: Ontario Health Insurance Plan
RPD: Registered persons database
IRCC-PR: Immigration Refugees and Citizenship Canada Permanent Resident
NHL: Non-Hodgkin lymphoma
HL: Hodgkin lymphoma
SD: Standardized difference
AMH: Anti-Müllerian hormone
AOF: Acute ovarian failure
