# Correction: Premature ovarian insufficiency in female adolescent and young adult survivors of non-gynecological cancers: a population-based cohort study

**DOI:** 10.1186/s12978-023-01579-y

**Published:** 2023-03-30

**Authors:** Sydney B. Flatt, Amanda Baillargeon, Chad McClintock, Jessica Pudwell, Maria P. Velez

**Affiliations:** 1 School of Medicine, Queen’s University, 15 Arch St., Kingston, ON K7L 3L4 Canada; 2 Department of Obstetrics and Gynecology, Queen’s University, 76 Stuart St., Victory 4, Kingston, ON K7L 2V7 Canada; 3 ICES Queen’s, 21 Arch St, Kingston, ON K7L 2V7 Canada

**Correction: Reproductive Health (2023) 20:4** **https://doi.org/10.1186/s12978-022-01559-8**

After publication of this article [1], the authors reported that the Acknowledgments section should have read as follows:

This study was supported by ICES, which is funded by an annual grant from the Ontario Ministry of Health (MOH) and the Ministry of Long-Term Care (MLTC). Parts of this material are based on data and information compiled and provided by the MOH. The analyses, conclusions, opinions, and statements expressed herein are solely those of the authors and do not reflect those of the funding or data sources; no endorsement is intended or should be inferred. Parts of this material are based on data and information provided by Ontario Health (OH). The opinions, results, view, and conclusions reported in this paper are those of the authors and do not necessarily reflect those of OH. No endorsement by OH is intended or should be inferred. Parts of this material are based on data and information compiled and provided by CIHI. The analyses, conclusions, opinions and statements expressed herein are solely those of the authors and do not reflect those of the funding or data sources; no endorsement is intended or should be inferred. Parts or whole of this material are based on data and/or information compiled and provided by Immigration, Refugees and Citizenship Canada (IRCC). However, the analyses, conclusions, opinions and statements expressed in the material are those of the author(s), and not necessarily those of IRCC. The authors would like to acknowledge Wenbin Li at ICES Queen’s for assistance with additional statistical analysis.

The original article [1] has been corrected.
